# Aclarubicin stimulates RNA polymerase II elongation at closely spaced divergent promoters

**DOI:** 10.1126/sciadv.adg3257

**Published:** 2023-06-14

**Authors:** Matthew Wooten, Brittany Takushi, Kami Ahmad, Steven Henikoff

**Affiliations:** ^1^ Fred Hutchinson Cancer Center, Seattle, WA 98109, USA.; ^2^ Howard Hughes Medical Institute, Chevy Chase, MD 20815, USA.

## Abstract

Anthracyclines are a class of widely prescribed anticancer drugs that disrupt chromatin by intercalating into DNA and enhancing nucleosome turnover. To understand the molecular consequences of anthracycline-mediated chromatin disruption, we used Cleavage Under Targets and Tagmentation (CUT&Tag) to profile RNA polymerase II during anthracycline treatment in *Drosophila* cells. We observed that treatment with the anthracycline aclarubicin leads to elevated levels of RNA polymerase II and changes in chromatin accessibility. We found that promoter proximity and orientation affect chromatin changes during aclarubicin treatment, as closely spaced divergent promoter pairs show greater chromatin changes when compared to codirectionally oriented tandem promoters. We also found that aclarubicin treatment changes the distribution of noncanonical DNA G-quadruplex structures both at promoters and at G-rich pericentromeric repeats. Our work suggests that the cancer-killing activity of aclarubicin is driven by the disruption of nucleosomes and RNA polymerase II.

## Teaser

Anti-cancer drug aclarubicin promotes RNA polymerase II elongation, chromatin accessibility and G-quadruplex formation.

## INTRODUCTION

Chromatin proteins shape the physical structure of DNA by regulating accessibility, torsion, and three-dimensional association (*1*–*3*). Nucleosomes, the fundamental unit of eukaryotic chromatin structure, are composed of two copies each of histones H3, H4, H2B, and H2A and wrap ~1.7 turns, or 146 base pairs (bp) of DNA (*4*). Nucleosomes present a formidable barrier to transcription initiation and elongation, and numerous auxiliary factors are required to remove or remodel nucleosomes to allow for efficient transcriptional initiation and progression. RNA polymerase II (RNA Pol II) dynamics associated with active transcription can also substantially affect chromatin structure. Transcription can lead to dynamic changes including histone turnover, the addition of posttranscriptional modification, and changes in histone variant composition (*5*). Transcription can also induce changes in DNA structure: unwound or negatively supercoiled DNA generated in the wake of the advancing polymerase can promote the formation of noncanonical DNA structures, such as G-quadruplexes (*2*, *6*), which are composed of G-rich DNA held together by Hoogsteen base-pairing arrangements (*7*).

Changes in chromatin structure can also be induced by small-molecule drugs that intercalate between DNA bases, such as anthracyclines, a class of anticancer drugs given to more than 1 million patients per year (*8*). Most anthracyclines used in the clinic also poison topoisomerases (*9*), which are enzymes responsible for relieving torsional strain on DNA (*10*). A popular model for the anticancer effect of anthracyclines is that by poisoning topoisomerase, anthracyclines generate DNA damage, leading to programmed cell death (*9*). However, this model has been challenged by studies demonstrating that anthracycline intercalation promotes nucleosome turnover and causes chromatin disruption (*8*, *11*–*16*). One particular anthracycline—aclarubicin—generates high levels of histone eviction without causing DNA damage, while the anthracycline doxorubicin causes only moderate levels of histone eviction but high levels of DNA damage (*13*). These differences have potential clinical implications, as chromatin disruption is better correlated with cancer cell death than DNA damage (*17*). However, it remains unclear how extensive anthracycline-induced chromatin disruption is, how this may differ between anthracyclines, and what the downstream impacts of chromatin disruption are on cellular functions such as transcription.

In this study, we investigated the impacts of anthracycline-mediated chromatin disruption on RNA Pol II in *Drosophila melanogaster* cells, a system where transcriptional regulation has been extensively characterized (*18*, *19*). We find that aclarubicin, but not doxorubicin, induces gains in both RNA Pol II and chromatin accessibility. We also find that distinct genomic regions show differential responses to aclarubicin treatment: closely spaced divergent promoter elements show greater increases in RNA Pol II and chromatin accessibility when compared to more distantly spaced promoter elements. We also show that aclarubicin treatment induces chromatin disruption at the pericentromeric Dodeca-satellite repeat blocks, which display gains in RNA Pol II, chromatin accessibility, and G-quadruplex formation following aclarubicin treatment. The distinct impacts that doxorubicin and aclarubicin have on chromatin suggest that the anticancer effects of these two anthracyclines are caused by distinct mechanisms.

## RESULTS

### Aclarubicin treatment inhibits proliferation and increases RNA Pol II in genes

**Fig. 1.**
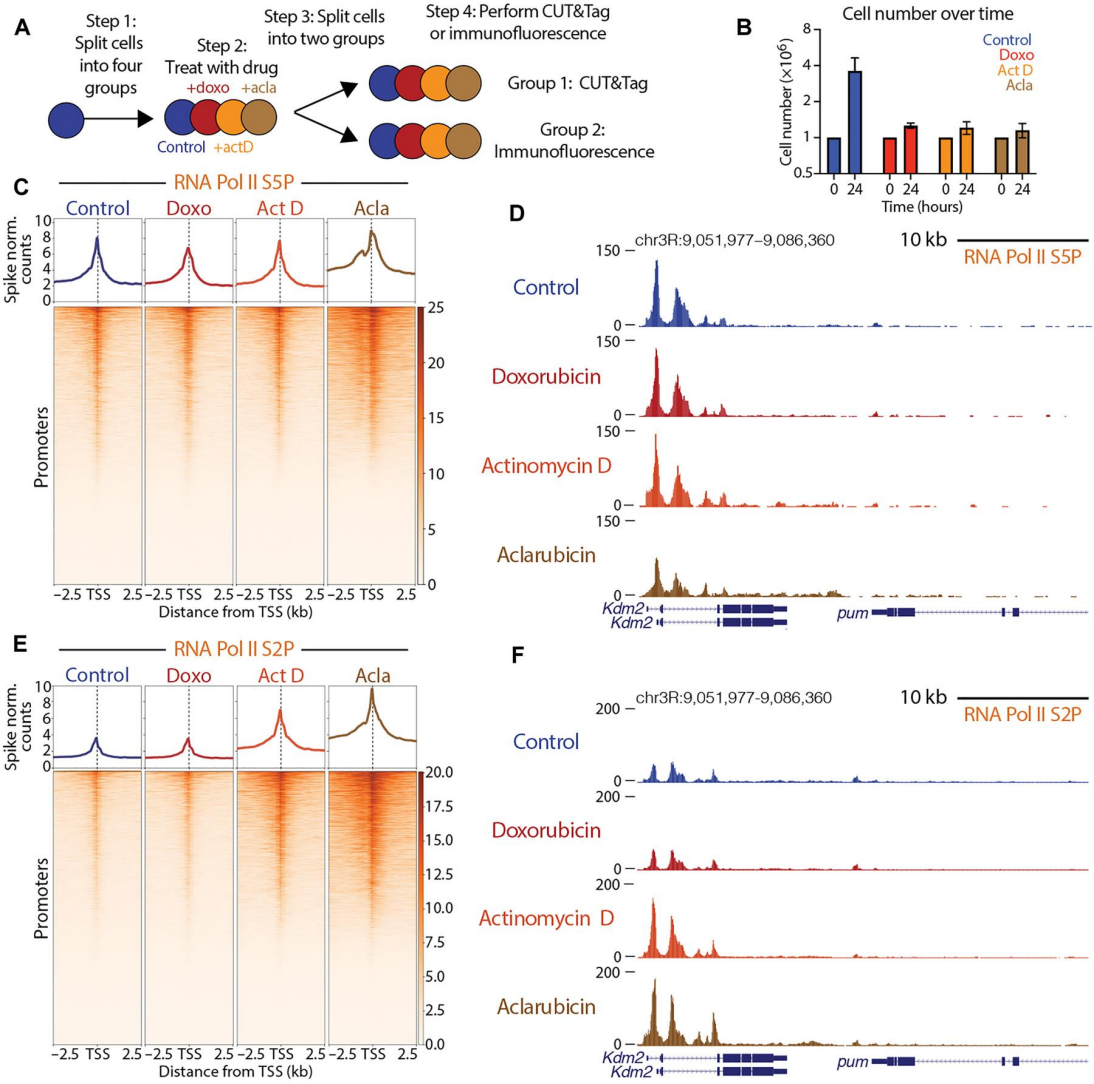
Aclarubicin blocks growth and causes gains in RNA Pol II Ser2P. (**A**) Experimental design. The four treatments were 1 μM doxorubicin (doxo), 5 μM actinomycin D (Act D), 1 μM aclarubicin (acla), and mock-treated dimethyl sulfoxide control. (**B**) Cell numbers at 0 and 24 hours; *n* = 3 biological replicates. Error bars signify SEM. (**C**) Heatmap aligned to transcription start site (TSS) of all promoters showing paused polymerase spike-normalized (norm.) signal under each treatment condition. (**D**) Representative UCSC browser track snapshot of paused RNA Pol II distribution. Merged data of five biological replicates for control, Act D-, and acla-treated samples and six biological replicates for doxo-treated samples. (**E**) Heatmap aligned to the TSS of all promoters showing spike-normalized elongating polymerase signal under each treatment condition. (**F**) Representative UCSC browser track snapshot of elongating polymerase distribution. Merged data of six biological replicates for doxo-, Act D- and acla-treated samples and five biological replicates for control samples were shown. CUT&Tag, Cleavage Under Targets and Tagmentation.

To assess the impacts of drug treatment on cell morphology and viability, *Drosophila* Kc167 cells were resuspended in media containing 1 μM doxorubicin, 1 μM aclarubicin, or 5 μM actinomycin D, an intercalating drug known to arrest RNA Pol II elongation in gene bodies (Fig. 1A). After 24 hours of drug treatment, all three drugs similarly inhibited cell proliferation compared to mock-treated controls (Fig. 1B and table S1). Previous studies have shown that anthracyclines can also affect nucleolar structure (*20*). Using antibodies to fibrillarin as a nucleolar marker, we observed nucleolar signal in 97% of doxorubicin-treated cells after 24-hour treatment, demonstrating that, at this dose, the nucleolus is not disrupted. In contrast, only 15% of aclarubicin-treated cells had nucleolar fibrillarin signal, indicating the disruption of ribosomal DNA (rDNA) transcription in most cells (fig. S1, A and D). Thus, aclarubicin is a more effective disruptor of nucleolar structure than doxorubicin, despite these drugs’ structural similarities. Similarly, only 11% of actinomycin D-treated cells showed nucleoli, indicating the disruption of rDNA transcription. Using antibodies to cleaved caspase as a marker of apoptosis, we observed that after 24-hour treatment, both aclarubicin- and actinomycin D-treated cells showed high levels of cell death compared to controls, whereas doxorubicin-treated cells showed little increase (fig. S1, B and E). Last, we assessed cell size after drug treatment. Cells treated with doxorubicin were slightly larger than control cells, whereas both aclarubicin- and actinomycin D-treated cells were smaller than control cells (fig. S1C and table S2). Measurements of nucleolar integrity, size, and death after 1 hour of drug treatment showed no substantial changes for any of the three drugs (fig. S1, A to C and table S3). Therefore, we performed all subsequent experiments after 30 min of drug exposure, before observable signs of toxicity, to assess immediate effects of drug treatment on chromatin.

Nucleosomes can block RNA Pol II access to DNA at promoters (*21*, *22*). Therefore, active promoters are remodeled to remove nucleosomes and facilitate the assembly of the transcriptional pre-initiation complex (*23*, *24*). As previous work has shown that anthracyclines stimulate nucleosome turnover at gene promoters, we sought to understand whether anthracycline treatment affects RNA Pol II dynamics. To assess changes in RNA Pol II, we treated cells with drugs for 30 min, and then performed Cleavage Under Targets and Tagmentation (CUT&Tag) (*25*) targeting both paused RNA Pol II [marked by serine-5 phosphorylation (Ser5P)] and elongating RNA Pol II [marked by serine-2 phosphorylation (Ser2P)] (*26*). All CUT&Tag experiments profiling RNA Pol II were quantified using a human cell posttreatment spike-in that enables direct comparisons of chromatin factor levels between different samples treated across a single experiment (*27*, *28*). To visualize the amounts and distribution of RNA Pol II, we generated spike-normalized coverage heatmaps at all 16,972 promoters in the *D. melanogaster* genome centered on their transcription start site (TSS). While paused RNA Pol II Ser5P showed minimal changes across all three drugs (Fig. 1, C and D), aclarubicin-treated samples gained large amounts of elongating RNA Pol II Ser2P at and around active promoters (Fig. 1, E and F). Samples treated with actinomycin D also gained a modest amount of elongating RNA Pol II signal, similar to previous observations (*29*). In contrast, elongating RNA Pol II Ser2P signal in doxorubicin-treated samples showed minimal changes (Fig. 1, E and F). The observation that aclarubicin but not doxorubicin increases RNA Pol II elongation reveals a fundamental difference in the mechanisms underlying chromatin disruption in these distinct anthracyclines.

The intercalation of actinomycin D into DNA can block RNA Pol II and stimulate hyperphosphorylation (Ser2P) of RNA pol II’s C-terminal domain (*30*, *31*). To determine whether changes observed in RNA Pol II Ser2P levels during aclarubicin treatment (Fig. 1, E and F) result from RNA Pol II hyperphosphorylation or from increased RNA Pol II occupancy, we profiled total RNA Pol II following drug treatment using an antibody targeting the RPB3 subunit of the RNA Pol II complex. To determine how drug treatment affects the levels of total RNA Pol II at promoters, we generated spike-normalized coverage heatmaps at all 16,972 promoters in the *D. melanogaster* genome centered on their TSS (fig. S1F). Aclarubicin-treated samples showed an increase in total RNA Pol II levels relative to controls. Actinomycin D-treated samples, by contrast, showed a decrease in total RNA Pol II levels. These data for actinomycin D are similar to previous studies demonstrating that actinomycin D-treatment can lead to decreases in total chromatin-bound RNA Pol II (*32*, *33*). Doxorubicin-treated samples showed little difference in total RNA Pol II levels at promoters when compared to controls. To understand how drug treatment affects the relative distribution of total RNA Pol II at promoters, we generated plots showing fold change in total RNA Pol II coverage centered on the TSS for each drug treatment compared to controls (fig. S1, G to I). These plots revealed that aclarubicin-treated samples gained RNA Pol II signal downstream of the TSS (fig. S1I), while actinomycin D-treated samples showed a relative increase in RNA Pol II occupancy upstream of the TSS (fig. S1H). Doxorubicin-treated samples showed minimal differences in total RNA Pol II levels compared to controls (fig. S1G). Together, the differing impacts on the levels and distribution of total RNA Pol II at promoters suggest that aclarubicin and actinomycin D affect RNA Pol II via distinct mechanisms.

### Aclarubicin promotes chromatin accessibility and G-quadruplex formation

We next wondered whether the increase in RNA Pol II caused by aclarubicin might affect chromatin accessibility and G-quadruplex formation, both of which are associated with changes in RNA Pol II activity (*34*, *35*). To assay chromatin accessibility changes during drug treatment, we treated cells with each drug for 30 min, and then performed Cleavage Under Targeted Accessible Chromatin (CUTAC) (*25*). CUTAC has an identical workflow to CUT&Tag, except that it uses low-salt tagmentation conditions while tethering pA-Tn5 to RNA Pol II. Whereas high-salt conditions direct tagmentation to either side of RNA Pol II, low-salt conditions drive the tagmentation of accessible DNA adjacent to RNA Pol II. This allows CUTAC to produce chromatin accessibility maps with low background that are indistinguishable from ATAC-seq (assay for transposase-accessible chromatin using sequencing), which uses a freely diffusing, untethered transposase to target and tagment accessible DNA (*25*). To assess changes in G-quadruplexes during drug treatment, we performed CUT&Tag using the BG4 antibody, which primarily targets G-quadruplex structures (*36*, *37*). CUT&Tag does not require fixation or sonication, making it ideal for profiling G-quadruplexes which may be disrupted by such harsh treatments. Previous studies have demonstrated that CUT&Tag using the BG4 antibody is effective in profiling G-quadruplex structures (*38*, *39*).

**Fig. 2.**
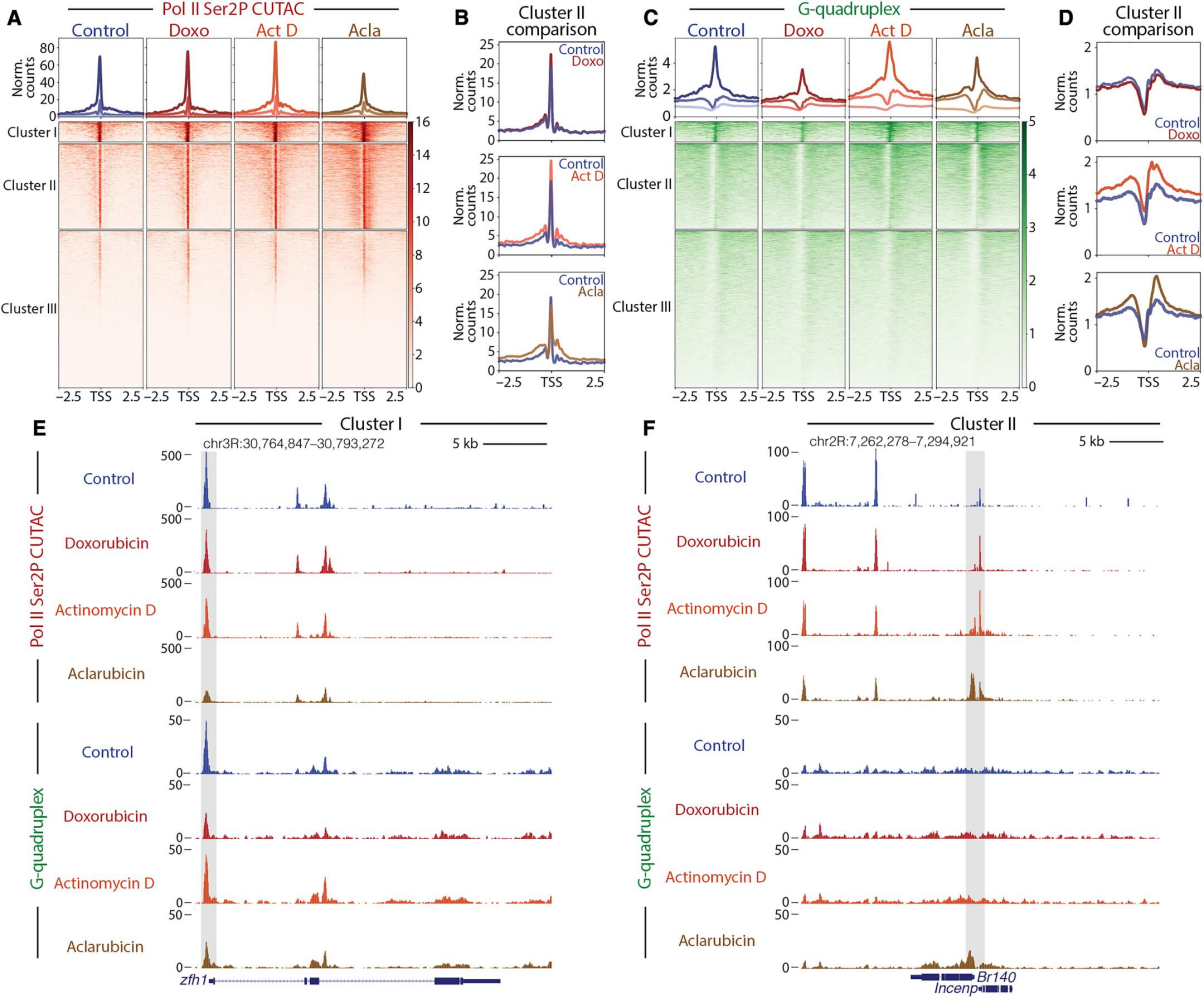
Drug treatment affects chromatin accessibility and G-quadruplex formation. (**A**) Heatmap aligned to TSS of all promoters showing normalized counts of CUTAC chromatin accessibility targeting RNA Pol II Ser2P clustered via k-means clustering (*k* = 3). (**B**) Enlarged comparison of accessibility differences between different drug groups and controls. Merged data of three biological replicates for Act D- and doxo-treated samples and two biological replicates for control and acla-treated samples were shown. (**C**) Heatmap aligned to the TSS of all promoters showing normalized counts of G-quadruplex CUT&Tag signal clustered via k-means clustering (*k* = 3). (**D**) Enlarged comparison of G-quadruplex differences between drug groups and controls. Merged data of three biological replicates for control, doxo-, and acla-treated samples and two biological replicates for Act D-treated samples were shown. (**E**) Representative UCSC browser track snapshot of CUTAC and G-quadruplex distribution at cluster I gene. The gray box indicates the promoter region. (**F**) Representative UCSC browser track snapshot of G-quadruplex distribution at cluster II gene. The gray box indicates the promoter region.

To visualize changes in chromatin accessibility during drug treatment, we performed k-means clustering of *Drosophila* promoters and generated heatmaps to compare accessibility across different groups (Fig. 2A). In control conditions, clusters I, II, and III are distinguished by levels of chromatin accessibility: Cluster I (*n* = 1306) promoters are highly accessible, cluster II (*n* = 5517) promoters are moderately accessible, and cluster III (*n* = 10,149) promoters display background levels of accessibility. Doxorubicin-treated samples showed minimal changes across all clusters, while actinomycin D-treated samples showed subtle gains in accessibility at regions proximal to the TSS (Fig 2, A, B, E, and F). Aclarubicin-treated samples, by contrast, showed reduced accessibility at cluster I promoters and increased accessibility upstream and downstream of the TSS at cluster II promoters (Fig. 2, A, B, E, and F).

We then used the same clusters generated by CUTAC data to assess changes in G-quadruplex formation between drug treatments. Doxorubicin-treated samples showed a loss of G-quadruplex signal at cluster I promoters, and no change in G-quadruplex signal at cluster II. Aclarubicin-treated samples showed a loss of G-quadruplex signal at cluster I but gains in G-quadruplex signal at cluster II promoters. Actinomycin D showed subtle gains in G-quadruplex signal across all promoter clusters, similar to previous reports demonstrating that actinomycin D can increase G-quadruplex formation at promoters (Fig. 2, C to F) (*39*). Regions showing the greatest gain in G-quadruplex signal during aclarubicin treatment were downstream of the TSS, distinct from the G-quadruplexes in cluster I that are located upstream of the TSS. The distinct spatial distribution of G-quadruplexes in cluster I and cluster II and their contrasting responses to aclarubicin treatment suggest that these G-quadruplexes may be formed and/or regulated in distinct ways.

Last, to assess changes in RNA Pol II at distinct gene clusters, we sorted RNA Pol II Ser5P and RNA Pol II Ser2P datasets using k-means clustering generated by CUTAC data. Similar to trends observed in CUTAC and G-quadruplex datasets, we observed that aclarubicin-treated samples showed gains in RNA Pol II Ser2P and RNA Pol II Ser5P at cluster II promoters (fig. S2, A to D and F). These gains in RNA Pol II signal were observable upstream and downstream of the promoter region (fig. S2, B and D). Actinomycin D-treated samples showed subtle gains in cluster I and cluster II, whereas doxorubicin-treated samples showed minimal changes across all clusters (fig. S2, A to F). Together, these data demonstrate that drug treatment differentially affects the distribution of RNA Pol II in distinct groups of genes.

### Closely spaced divergent promoters show greater chromatin disruption than distant promoters

**Fig. 3.**
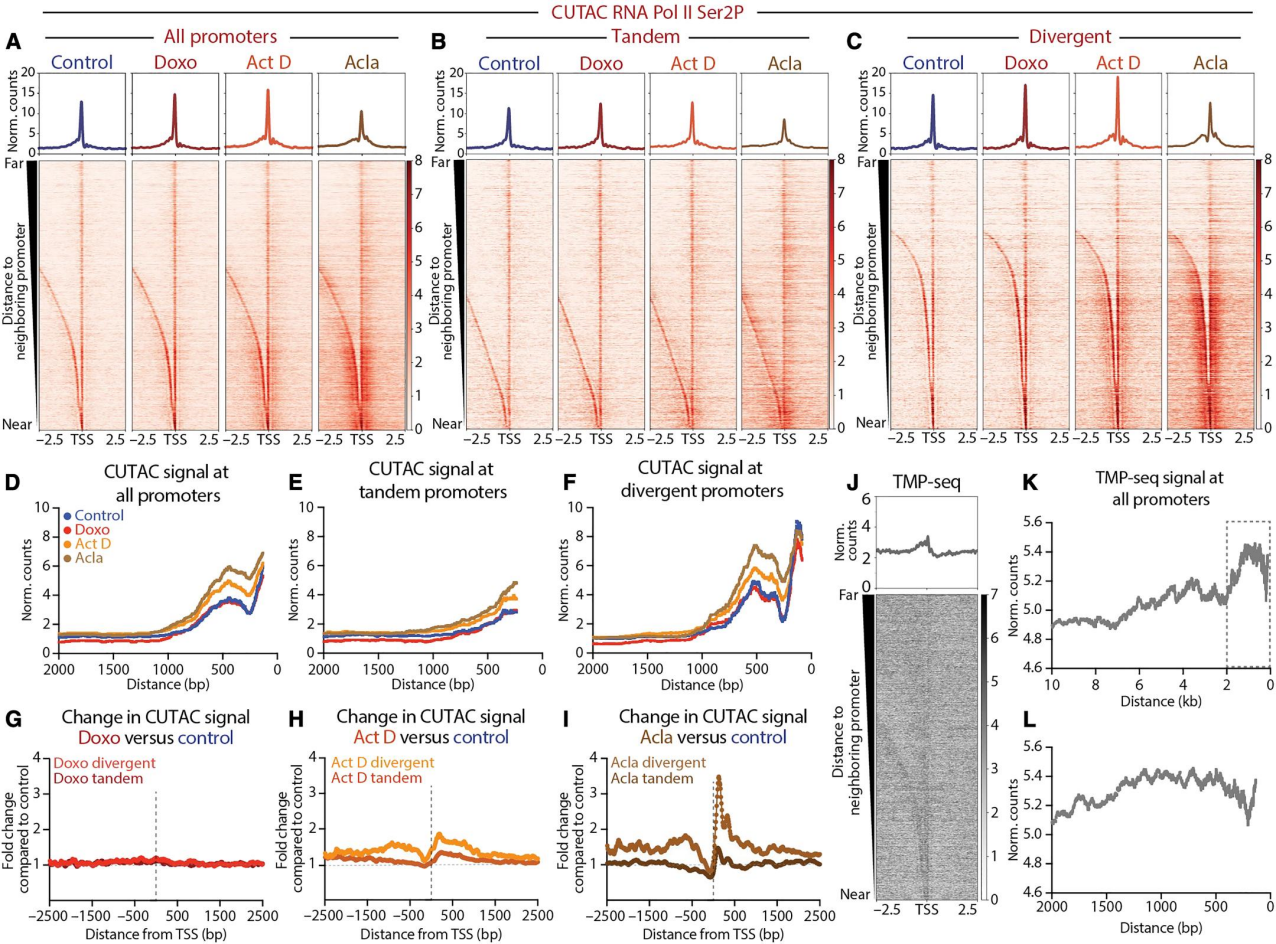
Promoter distance and orientation affect chromatin accessibility and superhelical torsion. Heatmaps of normalized counts showing CUTAC chromatin accessibility signal targeting RNA Pol II Ser2P aligned to the TSS of all promoters (**A**), tandem promoters (**B**), and divergent promoters (**C**) sorted by distance to the nearest upstream promoter element and plotted in descending order. (**D**) Plot showing moving median of CUTAC chromatin accessibility normalized counts as a function of distance between neighboring promoter elements for all promoters. (**E**) Plot showing moving median of CUTAC chromatin accessibility normalized counts as a function of distance between neighboring promoter elements for tandem promoters. (**F**) Plot showing moving median of CUTAC chromatin accessibility normalized counts as a function of distance between neighboring promoter elements for all divergent promoters. For (A) to (F), merged data of three biological replicates for Act D- and doxo-treated samples and two biological replicates for control and acla-treated samples were shown. (**G** to **I**) Plot showing fold change of CUTAC chromatin accessibility centered on the TSS for doxo versus control (G), Act D versus control (H), and acla versus control (I). The horizontal dotted line indicates no change compared to controls. The vertical dotted line indicates TSS. For (G), merged data of *N* = 3 doxo-treated samples were shown. For (H), merged data of three biological samples were shown. For (I), merged data from two biological replicates for acla-treated samples. (**J**) Heatmap of trimethylpsoralen sequencing (TMP-seq) data (*43*) aligned to the TSS of all promoters, sorted by distance to the nearest upstream promoter element, and plotted in descending order. (**K**) Plot showing the moving average of TMP-seq data as a function of the distance between neighboring promoter elements for all promoters. (**L**) Zoom in on the boxed region in (K).

We sought to understand what features differentiate genes in cluster I from genes in cluster II. While examining gene promoters in each cluster, we noticed that cluster I promoters are frequently isolated in the genome, whereas cluster II promoters are frequently found close to gene neighbors. To assess whether proximity to neighboring genes correlates with differential responsiveness to anthracyclines, we sorted promoters by their distance to the nearest upstream promoter and generated heatmaps of chromatin features. Closely spaced promoters in drug-treated samples appear to show greater gains in chromatin accessibility, G-quadruplex formation, and RNA Pol II Ser2P/5P than more distant promoters (Fig. 3A and fig. S3, A to C).

To better assess the quantitative differences in accessibility between closely spaced versus distant promoters, we plotted the CUTAC signal for each treatment as a function of the distance between promoters. All treatment groups showed increased accessibility at closely spaced promoter elements when compared to distantly spaced promoter elements (Fig. 3D and fig. S3D). Doxorubicin-treated samples showed minimal overall differences in accessibility when compared to control samples (Fig. 3D and fig. S3D). Quantification of accessibility for promoters with an inter-promoter distance less than 2 kb revealed a slight but statistically significant difference in accessibility for doxorubicin-treated samples (median = 2.78) when compared to controls (median = 2.64; fig. S3G). For promoters spaced greater than 2 kb apart, doxorubicin-treated samples showed lower accessibility values (median = 0.62) when compared to controls (median = 0.97; fig. S3G). Actinomycin D-treated samples showed no statistically significant difference for promoters greater than 2 kb apart (act D median = 1.10 versus control median = 0.97) and greater accessibility for promoters less than 2 kb apart (act D median = 3.58 versus control median = 2.64; Fig. 3D and fig. S3, D and G). Aclarubicin-treated samples showed the most notable differences in accessibility as a function of distance. While distantly spaced promoters showed no difference in accessibility when compared to control samples, closely spaced promoters showed significantly greater median accessibility relative to controls. This switch in accessibility appears to occur at ~1 kb (Fig. 3D and fig. S3D), where aclarubicin-treated samples first begin to show consistently higher accessibility. Quantification of median accessibility for closely spaced promoters (<2 kb) confirmed that aclarubicin-treated samples show significantly higher accessibility (acla median = 4.33 versus control median = 2.64), while distantly spaced promoters (>2 kb) show no significant difference (acla median = 0.96 versus control median = 0.97; fig. S3G). These results demonstrate that aclarubicin treatment disrupts chromatin structure most effectively at closely spaced promoters.

As closely spaced promoters show the greatest changes in chromatin structure during drug treatment, we wondered whether these promoters are transcribed in the same (tandem) or divergent directions. We found that 58% (5372 out of 9288) of the promoters less than 2 kb apart are divergently oriented, with that percentage growing to 68% (4604 out of 6788) for promoters positioned closer than 1 kb. To determine whether accessibility differed in closely spaced divergent promoters versus closely spaced tandem promoters, we separated these two groups of promoters and plotted the accessibility changes during drug treatment (Fig. 3, B, C, F, and G, and fig. S3, D to F). We observed that divergent promoters showed greater levels of accessibility overall and greater increases in accessibility following drug treatment when compared to tandem promoters. To further assess differences between divergent and tandem promoters across drug treatments, we plotted fold change in accessibility surrounding the TSS (Fig. 3, G to I) for each drug treatment. Whereas doxorubicin-treated samples showed minimal differences in accessibility when compared to controls (Fig. 3G), actinomycin D-treated samples showed modest increases in accessibility, with divergent promoters showing greater gains in accessibility compared to tandem promoters (Fig. 3H). Aclarubicin-treated samples showed the greatest increases in accessibility relative to controls, with divergent promoters once again showing the greatest fold change in accessibility (Fig. 3I).

As previous studies have shown that highly expressed genes are sensitive to anthracycline-mediated chromatin disruption (*14*), we next sought to determine whether differences in gene expression could underlie differences in chromatin disruption observed for divergent versus tandem promoter pairs during aclarubicin treatment (Fig. 3I). We sorted genes by promoter orientation and by expression, as defined by RNA sequencing (RNA-seq) (*40*). We found the median expression of divergent genes to be significantly higher than that of tandem promoters (median 210.3 versus 34.5 respectively; fig. S3H). We then grouped genes with divergent or tandem promoters into expression quintiles by RNA-seq, with quintile 1 (Q1) containing highly expressed genes and quintile 5 (Q5) containing lowly expressed genes (fig. S3I). For both divergent and tandem promoters, quintiles with higher levels of gene expression showed greater gains in chromatin accessibility during aclarubicin treatment (fig. S3, I to K). These results are consistent with previous studies showing that anthracycline-mediated chromatin disruption is positively correlated with gene expression (*14*). Divergent promoters showed greater gains in chromatin accessibility during aclarubicin treatment, even when compared to tandem promoters with comparable or greater median expression values (fig. S3, I to K). These data indicate that promoter orientation affects anthracycline responsiveness independent of differences in gene expression.

Previous work from our laboratory proposed that negative supercoiling (underwound DNA) generated in the wake of transcribing RNA Pol II facilitates the intercalation of anthracyclines into the genome, leading to chromatin disruption (*8*). As transcriptionally active promoters can propagate negative superhelical torsion more than 1-kb upstream of the TSS (*41*, *42*), promoters in close proximity may be affected by supercoiling generated at nearby promoters. To determine whether differences in negative supercoiling distinguish closely spaced promoters from isolated promoters, we used data profiling trimethylpsoralen (TMP) intercalation across the genome (*43*). As psoralen preferentially intercalates into negatively supercoiled DNA, this method (TMP sequencing, TMP-seq) maps the distribution of negative supercoiling genome-wide (*44*). We observed that closely spaced promoters showed higher levels of negative supercoiling when compared to distantly spaced promoters (Fig. 3, J to L). As negative supercoiling should facilitate the intercalation of anthracyclines (*45*), concentrated negative supercoiling upstream of closely spaced divergent promoters could enable drug binding, resulting in chromatin disruption. As chromatin structure around promoters is critical for regulating RNA Pol II dynamics, it is likely that loss of chromatin integrity at these sites would be highly impactful in driving changes in RNA Pol II elongation and gains in promoter-proximal chromatin accessibility.

### Histone genes and pericentromeric Dodeca-satellite repeats show distinct responses to drug treatment

**Fig. 4.**
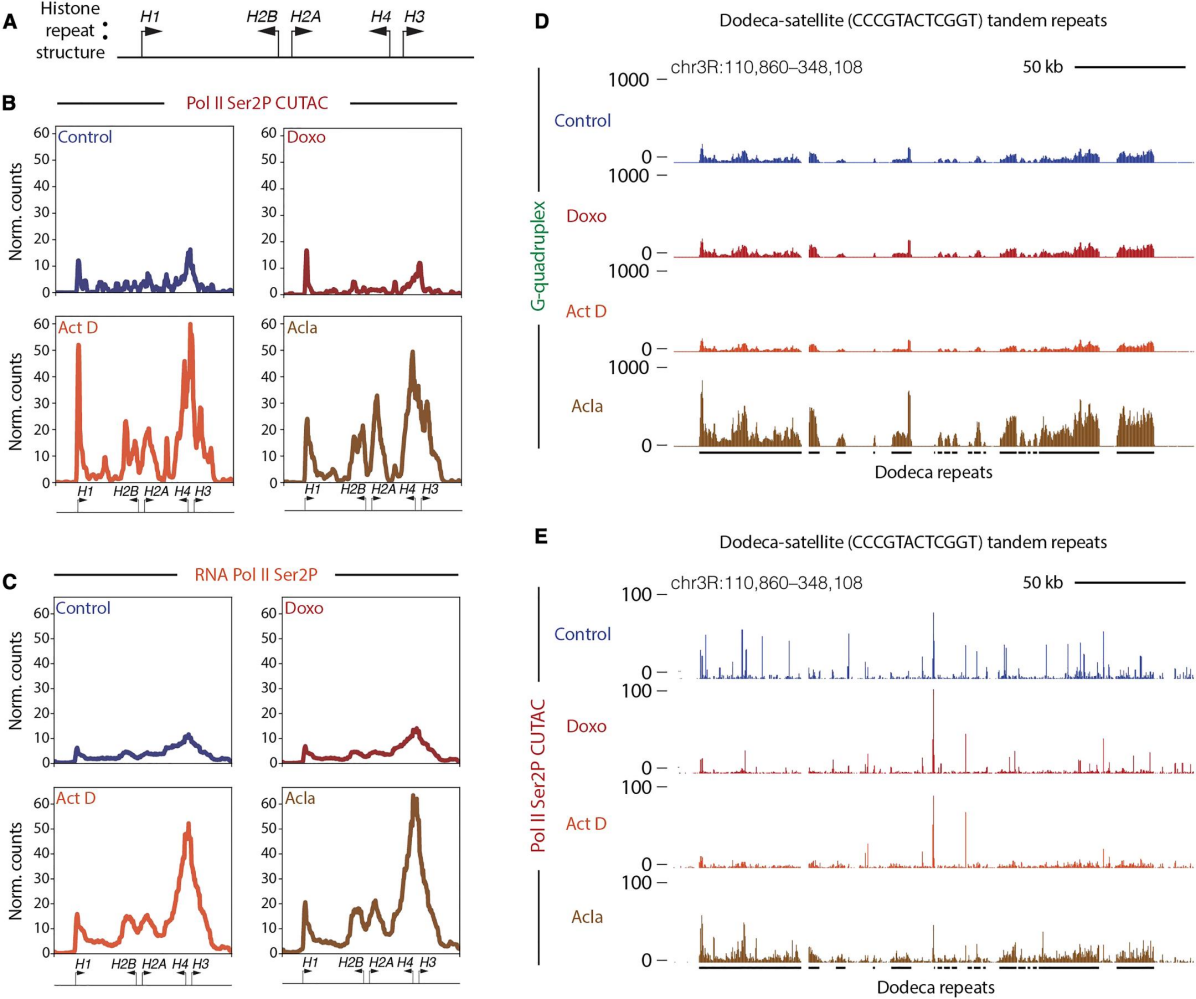
Histone cluster and Dodeca-satellite repeats show distinct responses to drug treatment. (**A**) Cartoon showing the divergent orientation of histone cluster genes. Arrows indicate the direction of transcription with the base of the arrow representing the approximate promoter position. (**B**) Average coverage plot of histone cluster showing normalized counts of CUTAC signal targeting RNA Pol II Ser2P. (**C**) Average coverage plot of histone clusters showing normalized counts of CUT&Tag data targeting RNA Pol II Ser2P. Arrows at the bottom of average plots indicate approximate positions of histone genes cartooned in (A). (**D**) UCSC browser track snapshot of G-quadruplex CUT&Tag data at Dodeca-satellite repeats. (**E**) UCSC browser track snapshot of RNA Pol II Ser2P CUTAC data at Dodeca-satellite repeats. Black lines below the browser tracks indicate the location of Dodeca-satellite repeats. Data for (A) and (E) are merged data of three biological replicates for Act D- and doxo-treated samples and two biological replicates for control and acla-treated samples. Data for (C) are merged data of six biological replicates for doxo-, Act D-, and acla-treated samples and five biological replicates for control samples. Data for (D) are merged data of three biological replicates for control, doxo-, and acla-treated samples and two biological replicates for Act D-treated samples.

The histone locus cluster is a region of the genome with an especially high incidence of closely spaced divergent promoters (Fig. 4A). Given the elevated levels of chromatin disruption observed at closely spaced divergent promoter elements genome-wide, we wondered whether the histone locus would also show changes in chromatin structure during anthracycline treatment. We found that, similar to divergent promoters genome-wide, the histone locus showed large gains in both chromatin accessibility (Fig. 4B) and elongating RNA Pol II (Ser2P) (Fig. 4C) following treatment with aclarubicin or actinomycin D. The histone locus did not show similar gains in initiating RNA Pol II Ser5P or G-quadruplex formation (fig. S4, A and B), further suggesting that the strongest immediate impacts of aclarubicin-mediated chromatin disruption are on elongating RNA Pol II. Since high levels of free histone proteins can induce cell death (*46*), this may contribute to the cytotoxicity of aclarubicin.

Previous studies have shown that the Dodeca-satellite (CCCGTACTCGGT) tandem repeats, found exclusively in a pericentromeric region of *D. melanogaster* chromosome 3, can form noncanonical structures known as i-motifs from the repetitive runs of cytosines found within the repeat (*47*). We wondered if G-quadruplexes form on the opposite strand from i-motif structures at Dodeca-satellite and if they are affected by drug treatment. We found that the G-quadruplex signal is detectable at Dodeca-satellite repeats in control datasets (Fig. 4D). Notably, G-quadruplex formation increased in cells treated with aclarubicin. This effect did not occur in cells treated with doxorubicin or with actinomycin D. Aclarubicin treatment also induced chromatin accessibility at the Dodeca-satellite repeats (Fig. 4E), as well as gains in RNA Pol II Ser2P and RNA Pol II Ser5P (fig. S4, C and D), while treatment with doxorubicin and actinomycin D did not. These data demonstrate that aclarubicin, but not doxorubicin or actinomycin D, is able to disrupt chromatin structure at this tandemly repetitive region of the genome.

## DISCUSSION

We have shown here that treatment of *Drosophila* cells with the anthracycline aclarubicin results in gains in RNA Pol II and changes in chromatin accessibility and G-quadruplex formation at active gene promoters. Closely spaced divergent promoter pairs are particularly sensitive to drug treatment, likely due to high levels of negative supercoiling enriched at these promoter elements. Chromatin effects are not limited to active gene promoters and can also be found at certain repetitive sequences prone to forming noncanonical DNA structures. Our observations reveal molecular features underlying anthracycline-mediated chromatin disruption and demonstrate clear differences between doxorubicin and aclarubicin treatment, suggesting that these drugs may exert anticancer effects through different mechanisms.

The predominant model for anthracycline cytotoxicity is that these drugs induce DNA damage by preventing topoisomerase II (topo II) from ligating together the double-strand breaks made during topoisomerase-mediated DNA cleavage (*48*). However, anthracyclines also disrupt chromatin structure by evicting histones (*13*) and increasing nucleosome turnover around promoters (*14*). Chromatin disruption is better correlated to cell death than DNA damage, suggesting that chromatin disruption is the principal anticancer activity of anthracyclines (*13*, *17*, *49*). Our results here demonstrate that aclarubicin, which is permissive for topo II ligation of double-strand breaks, promotes RNA Pol II elongation and chromatin accessibility at active promoters. Aclarubicin treatment leads to increased RNA Pol II in gene bodies and generates high levels of RNA Pol II Ser2P, implying that aclarubicin targets the conversion of initiating RNA Pol II into the elongating form. This effect may occur through aclarubicin-mediated disruption of the first nucleosome downstream of an active promoter. The first (+1) nucleosome is critical in regulating RNA Pol II elongation rates (*22*, *50*), and the disruption of the +1 nucleosome would remove a barrier to polymerase elongation. Thus, chromatin disruption and nucleosome turnover induced by aclarubicin have widespread effects on the transcriptional status of cells.

The potential for aclarubicin treatment to globally alter transcription may provide a functional bridge between chromatin disruption and the cancer-killing potential of anthracyclines. As many cancers are characterized by high levels of transcription (*51*) and transcriptional addiction (*52*), targeting global transcription has been proposed as a viable therapeutic strategy for treating cancer by disrupting the transcription programs needed to maintain cancer cell survival and proliferation (*53*). The disruption of transcription with small molecules such as actinomycin D has been shown to be effective in treating several cancers, including Ewing’s sarcoma, Wilm’s tumor, and rhabdomyosarcoma (*53*). Furthermore, studies have shown that loss of transcriptional control can lead to increased collisions between DNA replication machinery and RNA Pol II, which can lead to senescence (*54*) and cell death (*55*). Tight regulation of histone levels is also critical for cell survival, as low histone levels can lead to replication stress (*56*) and cell cycle arrest (*57*), while excess histones can drive elevated levels of chromosome loss and cell death (*46*). Furthermore, there is evidence to suggest that modulating histone levels is an effective strategy to cause cancer cell death (*58*). As our studies show that the histone locus is highly sensitive to aclarubicin-mediated chromatin disruption, it is conceivable that dysregulation of histone synthesis represents a potential anticancer feature of aclarubicin treatment.

G-quadruplexes have also been proposed as promising therapeutic targets for cancer treatment (*59*). G-quadruplexes are common in the promotor regions of many oncogenes (*60*), and studies have shown that stabilizing G-quadruplexes in key oncogenes such as myc and ras can lead to their eventual down-regulation (*59*, *61*). As many tumors are dependent on these genes for continued proliferation (*62*), loss of myc and/or ras could lead to loss of proliferative potential, senescence, and eventually programmed cell death (*63*). Furthermore, G-quadruplexes are a common feature in telomeric regions, and promoting G-quadruplex formation/stability in this context could disrupt telomere elongation, limiting the proliferative potential of cancerous cells (*59*).

Previous studies have shown that aclarubicin promotes higher levels of histone turnover than doxorubicin (*13*). These observations have prompted investigations into what molecular features of anthracyclines are responsible for driving histone disruption. Anthracyclines are tetracyclic molecules with an anthraquinone backbone connected by a glycosidic linkage to a sugar moiety (*17*). Recent studies have found that *N*,*N*-dimethylation of the carbohydrate appended to the anthraquinone aglycon is critical for both chromatin disruption and cytotoxicity, and the authors of this study speculated that this modification alters the interaction dynamics of anthracyclines with DNA (*17*). As previous studies have shown that doxorubicin-DNA aminal adducts can form between the 3′- NH2 of the doxorubicin sugar, the N2 of the guanine base, and formaldehyde (*64*, *65*), the addition of two methyl groups (as in the case of aclarubicin) to the critical amino sugar might convert aclarubicin from a covalent DNA intercalator into a reversible DNA intercalator, thereby affecting the dynamics by which aclarubicin perturbs the contacts between DNA and the surface of the nucleosome (*17*). A reversible intercalator may be able to disrupt chromatin structure without forming DNA adducts, which have been shown to inhibit processes such as RNA Pol II elongation (*66*). In this way, aclarubicin-mediated chromatin disruption could promote RNA Pol II elongation, whereas doxorubicin cannot. While it remains unclear what specific molecular features are responsible for aclarubicin’s distinct effect on chromatin structure, these studies emphasized that chemical modifications that increase chromatin damage are highly coordinated with cancer cell death, whereas chemical modifications associated with increased DNA damage are not (*13*, *17*, *48*, *49*).

Aclarubicin and doxorubicin also have distinct effects on topoisomerase activity (*67*). Doxorubicin blocks the catalytic enzymatic reaction of topo II and stabilizes an intermediate structure wherein cut DNA is covalently linked to the protein, thereby poisoning the enzyme (*11*). By contrast, aclarubicin acts as a catalytic inhibitor of topo II by blocking conformational changes in the enzyme (*68*) and preventing topo II from binding to DNA (*69*, *70*). Aclarubicin also acts as a topoisomerase I inhibitor (*67*, *71*). By inhibiting topo I and II without causing DNA damage, aclarubicin treatment likely generates high levels of negative supercoiling (*43*, *72*). As negative supercoiling facilitates the intercalation of anthracyclines into DNA (*45*), aclarubicin’s impact on topo I and II may promote its own binding and drive higher levels of intercalation, thereby resulting in greater chromatin damage than doxorubicin. High levels of negative supercoiling could also contribute to the gains in G-quadruplex formation observed in our studies, both directly by promoting the separation of the DNA duplex (*73*) or indirectly by promoting the formation of R-loops (*74*), which can help maintain strand separation and stabilize G-quadruplexes (*75*). As both R-loops (*76*) and G-quadruplexes (*77*) are refractory nucleosome formation, it is possible that the formation of noncanonical DNA structures underlies the gains in promoter-proximal chromatin accessibility observed during aclarubicin treatment.

It is notable that the chromatin effects of aclarubicin treatment are particularly severe at closely spaced divergent promoters. We showed that these promoters are enriched for negative supercoiling, likely due to the transcriptional activity of promoter pairs (*41*–*43*). Similar to the model described above, we speculate that increased negative supercoiling concentrated between closely spaced divergent promoters could drive greater drug binding, leading to greater chromatin damage. We also observed that highly expressed genes show greater chromatin disruption when compared to lowly expressed genes, similar to previous findings (*14*). As highly expressed genes require topo II to resolve transcription-coupled supercoiling (*41*, *78*), topo II inhibition by aclarubicin could lead to the accumulation of negative supercoiling, there facilitating drug entry into DNA. Promoters of highly transcribed genes are also associated with low nucleosome occupancy (*35*). As nucleosomal DNA is refractory to the intercalation of small molecules (*79*), it is likely that low nucleosome occupancy at highly expressed genes promotes drug entry into DNA, leading to chromatin disruption.

A recent study in breast cancer patient samples and cell lines revealed that the elevated expression of factors that promote chromatin accessibility and gene expression, such as COMPASS, BAF, KDM4B, and KAT6B, leads to increased sensitivity to anthracycline treatment, whereas factors that promote chromatin compaction, such as PRC2, lead to anthracycline resistance (*80*). These studies suggest that the chromatin environment could be playing a critical role in rendering cancer cells more susceptible to the influence of anthracyclines, possibly by increasing the accessibility of anthracyclines to DNA. These observations are in agreement with previous studies (*81*), which have proposed that instability in cancer cell chromatin (*81*–*83*) could be a factor in rendering cancer cells more susceptible to the impacts of intercalating drugs when compared to healthy cells (*81*).

The effects of aclarubicin are not limited to active gene promoters, as aclarubicin treatment is able to generate elevated levels of both RNA Pol II Ser2P and RNA Pol II Ser5P at Dodeca-satellite repeats. These sequences are not normally transcribed in somatic cells but can form noncanonical DNA structures (*47*). Drug intercalation may trigger misfolding of these DNAs, stimulating their transcription (*84*). Our results imply that aclarubicin broadly alters chromosome structure, which may contribute to its cytotoxic properties.

Other chemotherapeutic agents such as curaxins have also been shown to intercalate into DNA, drive nucleosome turnover, and induce aberrant DNA structures (*81*, *85*). Like anthracyclines, the amount of chromatin disruption caused by curaxins is correlated with cytotoxicity, whereas DNA damage is not (*85*). Thus, chromatin disruption may be a major mechanism of action for distinct chemotherapies. Such a model is encouraging, as DNA damage contributes to many undesirable off-target effects of anthracyclines, including cardiotoxicity and therapy-related tumors (*13*).

## MATERIALS AND METHODS

### Cell culture and drug treatment

*Drosophila* Kc167 cells (RRID:CVCL_Z834) were grown to log phase in HYQ-SFX Insect medium (Invitrogen) supplemented with 18 mM l-glutamine and harvested as previously described (*43*). All cell counts and measures of cell size were measured using the Vi-CELL XR Cell Viability Analyzer (www.beckman.com). Doxorubicin (Sigma-Aldrich, D1515-10MG), aclarubicin (Cayman Chemical Company, 15993), and actinomycin D (Sigma-Aldrich, A9415-2MG) were resuspended to 10 mM in dimethyl sulfoxide and frozen in aliquots. For cell treatments, compounds were added to a cell medium containing 1.5 × 10^6^ cells per ml to a final concentration of 1 μM (aclarubicin and doxorubicin) or 5 μM (actinomycin D) and incubated at room temperature (RT). Cells were harvested after 30 min for imaging and CUT&Tag profiling, or 1 hour or 24 hours to assess growth rates and cell size, cell death, and nucleolus structure.

### Immunofluorescent labeling

Samples of 500,000 Kc167 cells were harvested, spun down, and resuspended in ice-cold phosphate-buffered saline (PBS) with 0.1% Triton X-100 (PBST) and 0.1% formaldehyde and incubated for 10 min on ice. Cells were spun down and resuspended in ice-cold PBST. Cells were then spun down onto a clean glass slide (Fisherbrand Superfrost Plus Microscope Slides; ThermoFisher Scientific, catalog no. 12-550-15) using a Thermo Scientific Shandon Cytospin 4. Slides were washed twice with 50 ml of 1× PBS each time and placed in a humid chamber with 1 ml of blocking solution (2.5% bovine serum albumin in 1× PBST) for 30 min of pre-blocking. The blocking buffer was then drained, and primary antibodies were added for incubation overnight at 4°C. Slides were then washed twice with 50 ml of 1× PBS and incubated with secondary antibodies for 2 hours at RT. Slides were then washed twice with 50 ml of 1× PBS and mounted with ProLong Diamond Mounting Media (Thermo Fisher Scientific, P36965). Cells were imaged on an EVOS auto 2.0 microscope (Thermo Fisher Scientific).

### Whole-cell CUT&Tag for chromatin profiling

CUT&Tag was performed as described (*27*, *86*) with some modifications. Briefly, two million *Drosophila* Kc167 cells were spun down and resuspended in 1 ml of ice-cold PBS containing 12,000 human K562 cells for spike-in normalization. Cells were spun down and resuspended in 1.5 ml of wash buffer [20 mM Hepes at pH 7.5, 150 mM KCl, 0.5 mM spermidine, and 1× protease inhibitors] by gentle pipetting. Five microliters of concanavalin A–coated magnetic beads (Smart-Lifesciences) were activated and added to cells and incubated for 10 min on ice. The supernatant was then removed, and bead-bound cells were resuspended in 100 μl of dig-wash buffer [20 mM Hepes at pH 7.5, 150 mM KCl, 0.5 mM spermidine, 1× protease inhibitors, and 0.05% digitonin] containing 2 mM EDTA and a 1:25 dilution of the BG4 primary antibody (0.1 mg/ml) (Sigma-Aldrich: MABE917). Primary antibody incubation was performed overnight at 4°C and then the liquid was removed. Cells were washed 3× in 200 μl dig-wash buffer. The wash buffer was then drained, and cells were resuspended in 100 μl of dig-wash buffer containing 2 mM EDTA and a 1:50 dilution of mouse anti-FLAG antibody (Sigma-Aldrich; F1804-1MG; RRID:AB_262044) and incubated at RT for 1 hour. Cells were washed three times in 200 μl dig-wash buffer. The wash buffer was then drained, and cells were resuspended in 100 μl dig-wash buffer containing 2 mM EDTA and a 1:50 dilution of rabbit anti-mouse antibody (Sigma, M7023) and incubated at RT for 1 hour with slow rotation. A 1:50 dilution of pA-Tn5 adapter complex was prepared in dig-300 buffer [0.05% digitonin, 20 mM Hepes at pH 7.5, 300 mM KCl, 0.5 mM spermidine, and 1× protease inhibitors). Fifty microliters of pA-Tn5 adapter complex was then added to the cells with gentle vortexing, followed by incubation for 1 hour at RT. Cells were washed three times in 200 μl of dig-300 buffer to remove unbound pA-Tn5 proteins. Cells were then immersed in 100 μl of tagmentation buffer (dig-300 buffer with 10 mM MgCl_2_) and incubated at 37°C for 1 hour. Cells were placed on a magnet and the supernatant was removed. Cells were washed with 50 μl of 10 mM N-[Tris(hydroxymethyl)methyl]-3-aminopropanesulfonic acid (TAPS) with 16.5 mM EDTA and resuspended in 100 μl of buffer containing 10 mM TAPS, 16.6 mM EDTA, 0.1% SDS, and proteinase K (0.2 mg/ml) and incubated at 56°C for 1 hour. Two hundred microliters of 300 wash buffer [20 mM Hepes at pH 7.5, 300 mM NaCl, 0.5 mM spermidine, and 1× protease inhibitors] was then added to the tube, after which, the DNA was extracted via phenol-chloroform isoamyl alcohol (PCI) for library preparation. Twenty-one microliters of DNA was mixed with a universal i5 and a uniquely barcoded i7 primer and amplified with NEB Q5 high-fidelity 2× master mix (catalog no. M0492S). The libraries were purified with 1.1× volume of Sera-Mag carboxylate-modified magnetic beads and subjected to LabChip DNA analysis and Illumina sequencing. A similar methodology was used for profiling elongating RNA Pol II (anti-RNAPII Ser2-phosphorylation antibody; Cell Signaling Technology, catalog no. 13499S), initiating RNA Pol II (anti-RNAPII Ser5-phosphorylation antibody; Cell Signaling Technology, catalog no. 13523 s), and total RNA Pol II (Anti-Rpb3; Bethyl Laboratories, catalog no. A303-771A) using 80,000 *D. melanogaster* whole cells. In some experiments, nuclei were extracted and profiled according to the CUT&Tag-direct protocol (*25*). CUTAC was performed as described using an antibody to RNA Pol II Ser2 phosphorylation (*87*).

### CUT&Tag data processing and analysis

Libraries were sequenced on an Illumina HiSeq instrument with paired-end 50 × 50 reads. Adapters were clipped by cutadapt (http://dx.doi.org/10.14806/ej.17.1.200) version 2.9 with the following parameters:

-j 8 --nextseq-trim 20 -m 20 -a AGATCGGAAGAGCACACGTCTGAACTCCAGTCA -A AGATCGGAAGAGCGTCGTGTAGGGAAAGAGTGT -Z. Clipped reads were aligned by Bowtie2 (*88*) to the UCSC *D. melanogaster* Dm6 reference sequence (*89*) with the following parameters:

--very-sensitive-local --soft-clipped-unmapped-tlen --dovetail --no-mixed --no-discordant -q --phred33 -I 10 -X 1000. Clipped reads were also aligned by Bowtie2 (*88*) to the UCSC *Homo sapiens* HG19 reference sequence (*89*) with the following parameters: --end-to-end --very-sensitive --no-overlap --no-dovetail --no-mixed --no-discordant -q --phred33 -I 10 -X 1000. Properly paired reads were extracted from the alignments by SAMTools (version 1.9) (*90*). Spike-in calibrated *D. melanogaster* tracks in bigwig format were made by bedtools (*91*) 2.30.0 genomecov with a scaling factor of (10,000/number of fragments mapped to *H. sapiens*). Normalized count tracks in bigwig format were also made by bedtools (*91*) 2.30.0 genomecov which are the fraction of counts at each base pair scaled by the size of the reference sequence (137,567,484) so that if, the scaled counts were uniformly distributed, there would be 1 at each position. Spike-in calibrated bigwig files were then uploaded to Galaxy (*92*) and heatmaps were generated using the computematrix function in deepTools (version 3.5.1) (*93*). A bedfile containing a list of all *D. melanogaster* promoters was used by computematrix function to compare spike-normalized reads from bedgraph files aligned at all *D. melanogaster* promoters. The output of the computematrix function was visualized using the plotHeatmap function in Galaxy.

For k-means clustering analysis, we used the plotHeatmap function in Galaxy with k = 3 on RNA Pol II Ser2P CUTAC data. We then used this sorting for other datasets (G-quadruplex, RNA Pol II Ser2P CUT&Tag, and RNA Pol II Ser5P CUT&Tag) by using the bedfile output of the plotHeapmap function as the “regions to plot” file for subsequent computeMatrix operations.

For analysis of nearby promoters, the inter-promoter distance was calculated using promoter coordinates from the eukaryotic promoter database (https://epd.epfl.ch//index.php) (*94*). For each promoter in the database, the distance to the nearest promoter upstream was used to assign a distance value (in base pairs) to each promoter in the database. The strand information for each promoter was used to classify the nearest upstream promoter as a tandem (same strand) or divergent (opposite strand). Promoters with the nearest promoter on the opposite strand were classified as divergent, whereas promoters with the nearest promoter on the same strand were classified as tandem.

To generate plots showing changes in the CUTAC signal as a function of the distance between promoters (Fig. 3, D to I), the total CUTAC signal at each promoter from 100-bp upstream to 500-bp downstream was summed using the multiBigwigSummary function from Galaxy deepTools. These values were calculated for all promoters across control, doxorubicin-treated, actinomycin D-treated, and aclarubicin-treated conditions. Promoters were then sorted by inter-promoter distance from greatest to least. A moving median value was then calculated to assess changes in accessibility with decreasing inter-promoter distances. We chose to calculate the median accessibility for each decile or 10th of promoters with the closest inter-promoter distances. As there are 16,972 promoters in the Eukaryotic Promoter Database, 1697 represents 1/10th, or a decile of all promoters. Therefore, for each promoter, we calculated the median CUTAC values for 1697 promoters with closest inter-promoter distances (848 promoters with greater inter-promoter distance, 848 promoters with lesser inter-promoter distance). We performed a similar calculation on the distance between promoters to give a moving median value of inter-promoter distance for each data point plotted on the graph. We then plotted the median CUTAC values as a function of the median distance. To generate plots showing TMP-seq as a function of distance, we calculated the total TMP-seq signal 200-bp upstream of the TSS. This region was chosen, as this specifically looks at the negative supercoiling upstream of the TSS at promoter regions. We then performed a similar moving average using deciles as described above. Two values were removed from decile analysis as they were extreme outliers: tx_1 and MOEH_1 had TMP-seq values greater than 64 SDs above the mean (mean = 5.21, stdev = 5.25 tx_1 = 846.79; MOEH_1 = 344.57).

To generate plots shown in fig. S1 (G to I) comparing relative total RNA Pol II levels between drug-treated and control conditions, the computeMatrix function of Galaxy was used to generate coverage plots of normalized read counts centered on the TSS. The values of these coverages were exported using the “save the matrix values underlying the heatmap” option. These matrices contained normalized counts for each promoter broken up into 10-bp bins. We then generated average normalized counts values for each drug treatment group for every 10-bp bin up to 100-bp upstream and 500-bp downstream of the TSS. Average values at each 10-bp bin were then divided (drug value/control value) and the log_2_ value was taken to generate a log-fold-difference ratio plot surrounding the TSS.

To generate plots shown in Fig. 3 (E to G) comparing divergent and tandem promoters in control samples to divergent and tandem promoters in drug-treated samples, the matrix values underlying the heatmaps in Fig. 3 (B and C) were exported from Galaxy using the computeMatrix function. These matrices contained normalized counts for each promoter broken up into 10-bp bins. We then generated average normalized counts values for each drug treatment group and for each promoter orientation (divergent versus tandem) for every 10-bp bin up to 2500 bp surrounding the TSS. Average values at each 10-bp bin were then divided (drug value/control value) to generate a fold-difference ratio plot surrounding the TSS.

To generate plots shown in fig. S3 (H and I), RNA-seq values from Cherbas *et al*. (*40*) were downloaded and uploaded to Galaxy. The computeMatrix function was then used with the “save the matrix values underlying the heatmap” option selected to generate matrices of RNA-seq values centered at the TSS for genes with divergent- and tandemly oriented promoters. The values exported from Galaxy were then plotted on a log_10_ scale and a constant value of 1 was added to all genes to make genes with an RNA-seq value of 0 visible on the plot. The plotHeatmap function was then used to plot RNA-seq data in descending order, with the highest expressed genes at the top and the lowest expressed genes at the bottom. These lists were then exported from Galaxy and broken up into descending fifths to generate quintiles of highly expressed genes (Q1) and lowly expressed genes (Q5). These lists were then used in the computeMatrix function with the “save the matrix values underlying the heatmap” option selected to plot RNA-seq values for each quintile (Q1 to Q5) and for each promoter class (tandem versus divergent). To generate the plots for fig. S3 (J and K), the RNA-seq quintile lists were used with the computeMatrix function with the “save the matrix values underlying the heatmap” option selected to generate matrices containing CUTAC data for aclarubicin and control samples. These matrices contained normalized counts for each promoter broken up into 10-bp bins. We then generated average normalized counts values for each drug treatment group, for each quintile, and for each promoter orientation (divergent versus tandem) for every 10-bp bin up to 100-bp upstream and 500-bp downstream of the TSS. Average values at each 10-bp bin were then divided (aclarubicin value/control value) to generate a fold-difference ratio plot surrounding the TSS for each quintile sorted by RNA-seq values.

## Supplementary Materials

### This PDF file includes:

Figs. S1 to S4 Tables S1 to S3 Legends for supplementary excel files S1 to S4

### Other Supplementary Material for this manuscript includes the following:

Supplementary excel files S1 to S4

## Notes

View/request a protocol for this paper from *Bio-protocol*.
