# Supplementary material for: Aclarubicin stimulates RNA polymerase II elongation at closely spaced divergent promoters
Source: Sci Adv. 2023 Jun 14;9(24):eadg3257. doi: 10.1126/sciadv.adg3257 (PMC10266722; doi:10.1126/sciadv.adg3257)
Supplement: Supplementary file 1 — Figs. S1 to S4 Tables S1 to S3 Legends for supplementary excel files S1 to S4 [file sciadv.adg3257_sm.pdf]

Science Advances
AAAS

Supplementary Materials for

Aclarubicin stimulates RNA polymerase II elongation at closely spaced divergent promoters

Matthew Wooten *et al.*

Corresponding author: Steven Henikoff, steveh@fredhutch.org

Sci. Adv. **9**, eadg3257 (2023)
DOI: 10.1126/sciadv.adg3257

This PDF file includes:

Figs. S1 to S4
Tables S1 to S3
Legends for supplementary excel files S1 to S4

Other Supplementary Material for this manuscript includes the following:

Supplementary excel files S1 to S4

Supplementary Materials:

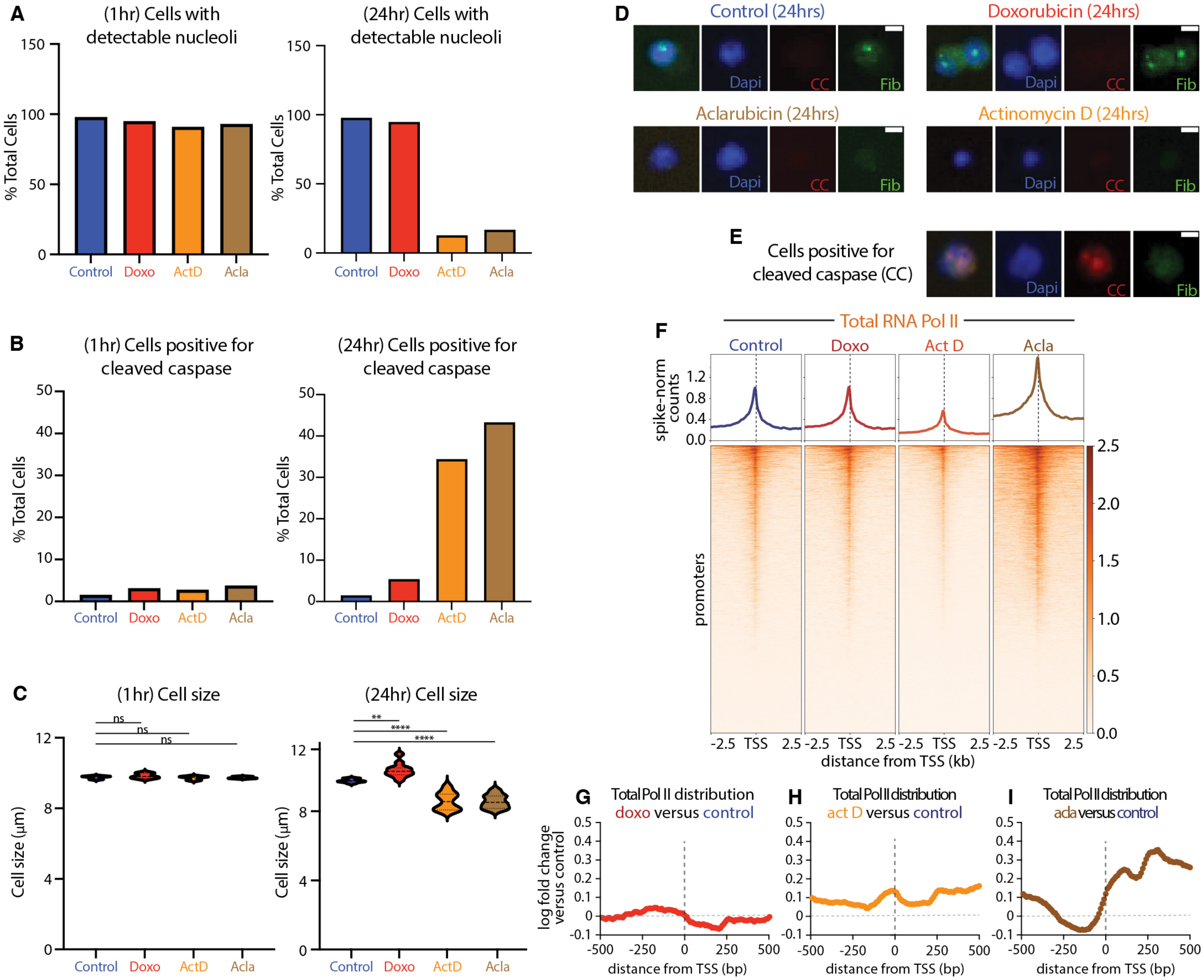

Fig. S1. Impacts of drug treatment after 1 hour and 24 hours and total RNA Pol II profiling at 30 minutes (**A**) Percent of cells with detectable nucleolus after 1 hour and 24 hours treatment. N = 100 cells for each treatment. (**B**) Percent of cells with detectable cleaved-caspase (cell death) staining after 1 hour and 24 hours treatment. Control 1 hour n = 129 cells, doxorubicin 1 hour n = 147 cells, actinomycin D 1 hour n = 123 cells, aclarubicin 1 hour n= 194 cells. Control 24 hours n = 126 cells, doxorubicin 24 hours n = 90 cells, actinomycin D 24 hours n = 81 cells, aclarubicin 24 hours n = 69 cells. (**C**) Cell size measurements after 1 hour and 24 hours treatment. n = 8 biological replicates for control, actinomycin D, aclarubicin; 11 biological replicates for doxorubicin. (**D**) Immunofluorescent images of DAPI, cleaved caspase (CC) and fibrillarin (nucleolar marker) after 24 hours treatment. (**E**) Example of cleaved caspase-positive cell. **(F)** Heatmap aligned to TSS of all promoters showing total RNA Pol II spike-normalized signal under each treatment condition. (**G-I**). Plots showing fold change in normalized counts of total RNA Pol II coverage for each drug treatment group *vs* control (drug/control): (**G**) doxorubicin *vs* control, (**H**) actinomycin D *vs* control and (**I**) aclarubicin *vs* control. Merged data of 3 biological replicates. Horizontal dotted line indicates no relative change from control. Vertical dotted line indicates TSS. Scale bar = 5µm. *ns* = not significant, **P<0.01; **** P<0.0001. Ordinary one-way ANOVA with Dunnett's multiple comparisons to control test. Violin plots for (C) indicate median, minimum, maximum, first quartile and third quartile.

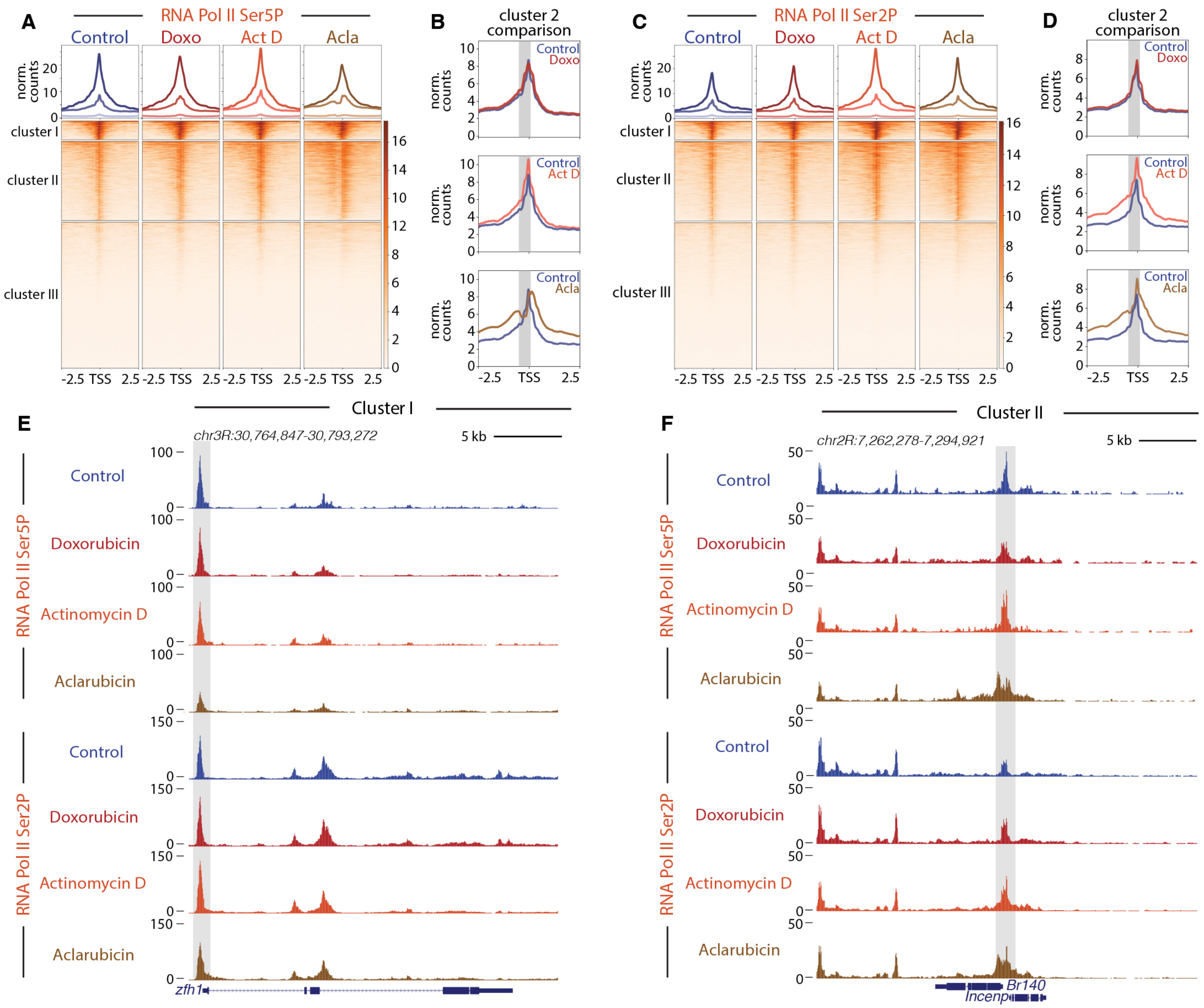

Fig. S2. Drug treatment impacts the relative distribution of RNA Pol II Ser5P and Ser2P (**A**) Heatmap aligned to the transcriptional start site (TSS) of all promoters showing normalized counts of RNA Pol II Ser5P CUT&Tag signal clustered via k-means clustering derived from CUTAC datasets (k=3). (**B**) Enlarged comparison of RNA Pol II Ser5P between different drug groups and controls. Gray box marks upstream promoter region. Merged data of 5 biological replicates for control, actinomycin D- and aclarubicin-treated samples and 6 biological replicates for doxorubicin-treated samples. (**C**) Heatmap aligned to TSS of all promoters showing normalized counts of RNA Pol II Ser2P CUT&Tag signal clustered via k-means clustering (k=3) derived from CUTAC datasets. (**D**) Enlarged comparison of RNA Pol II Ser2P differences between different drug groups and controls. Merged data of 6 biological replicates for doxorubicin-, actinomycin D- and aclarubicin-treated samples and 5 biological replicates for control samples. Gray box marks upstream promoter region. (**E**) Representative UCSC browser track snapshot of RNA Pol II Ser5P and RNA Pol II Ser2P distribution at a Cluster I gene. (**F**) Representative UCSC browser track snapshot of RNA Pol II Ser5P and RNA Pol II Ser2P distribution at a Cluster II gene.

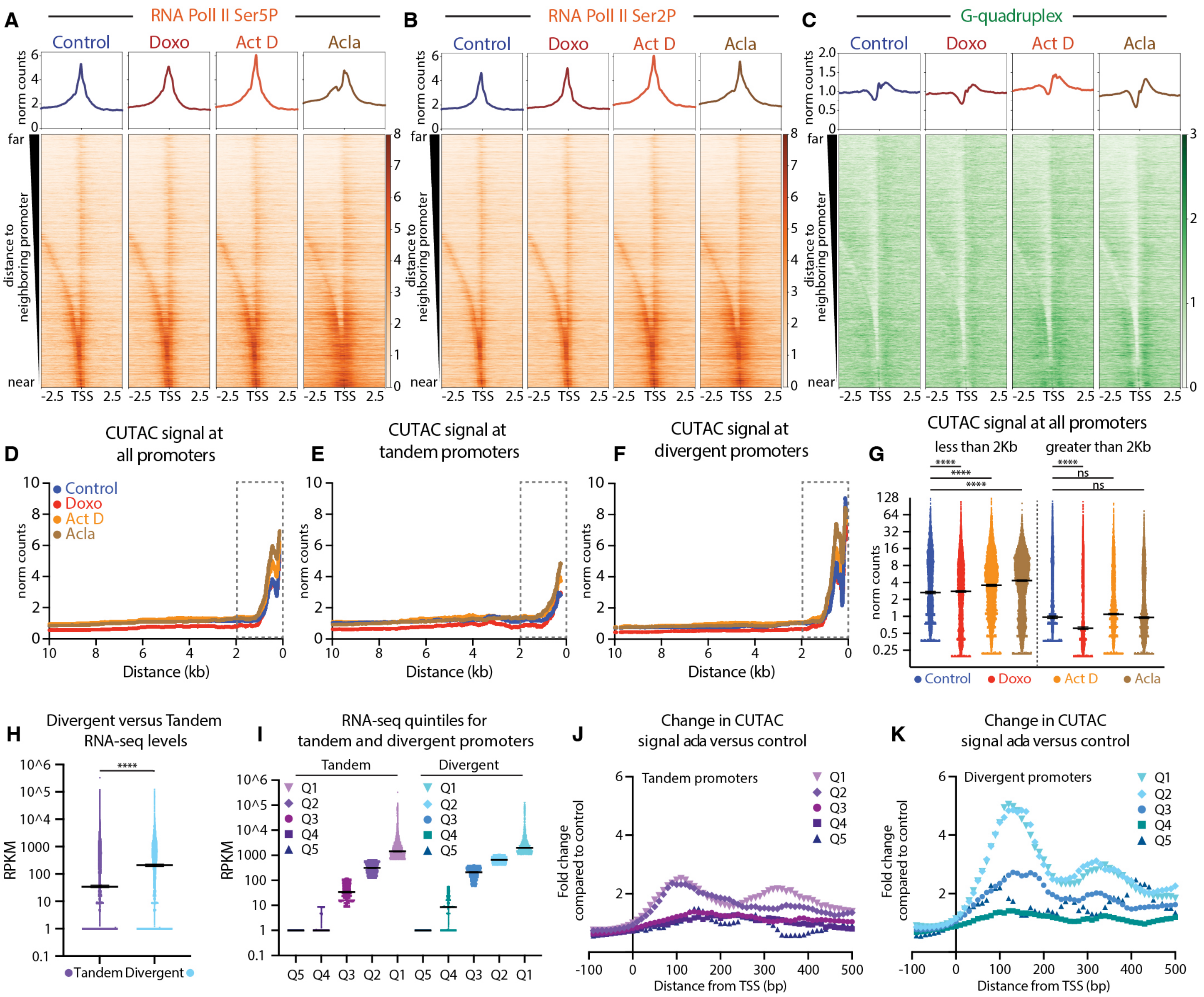

Fig. S3. Promoter distance and orientation affects RNA Pol II, G-quadruplexes, and accessibility. Heatmaps of CUT&Tag data targeting (**A**) RNA Pol II Ser5P, (**B**) RNA Pol II Ser2P and (**C**) G-quadruplexes aligned to the TSS of all promoters sorted by distance to nearest upstream promoter element and plotted in descending order. (**D-F**) Plot showing moving median of CUTAC chromatin accessibility normalized counts up to 10kb between neighboring promoter elements for all promoters (**D**), tandem promoters (**E**), and divergent promoters (**F**). (**G**) Plot showing CUTAC normalized counts for promoters less than 2kb apart and greater than 2kb apart. (**H**) Plot showing RNA-seq data in reads per kilobase per million mapped reads (RPKM) (*40*) for tandem *versus* divergent promoters (**I**) Plot showing RPKM for tandem *versus* divergent promoters broken up into ranked quintiles. (**J-K**). Plot showing fold change (aclarubicin/control) in CUTAC signal for tandem promoters (**J**) and divergent promoters (**K**) sorted by quintiles defined by ranked RNA-seq values shown in **I**. Median shown as black bar with 95% confidence interval. **** = p< 0.0001 Kruskal-Wallis test with multiple comparisons for panel G. Mann-Whitney test for panel H. Data for (A) is merged data of 5 biological replicates for control, actinomycin D- and aclarubicin-treated samples and 6 biological replicates for doxorubicin-treated samples. Data for (B) is merged data of 6 biological replicates for doxorubicin, actinomycin D- and aclarubicin-treated samples and 5 biological replicates for control samples. Data for (C) is merged data of 3 biological replicates for control, doxorubicin- and aclarubicin-treated samples and 2 biological replicates for actinomycin D-treated samples. Data for D-G, J-K is merged data from 3 biological replicates for actinomycin D-, doxorubicin-treated samples and 2 biological replicates for control and aclarubicin-treated samples.

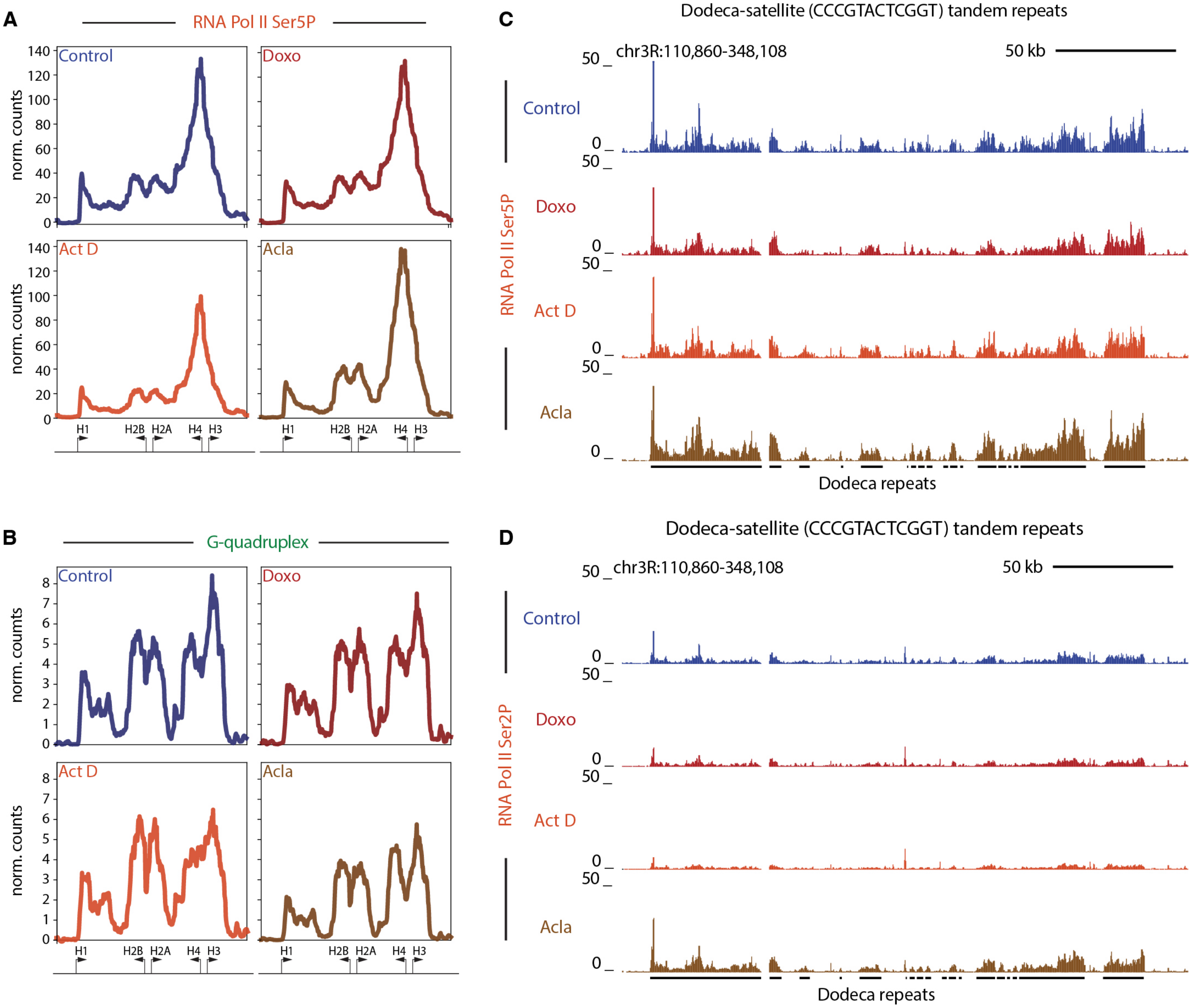

Fig. S4. Histone cluster and Dodeca-satellite repeats show distinct responses to drug treatment (**A**) Average coverage plot of histone clusters showing CUT&Tag data targeting RNA Pol II Ser5P. (**B**) Average coverage plot of histone clusters showing CUT&Tag data targeting G-quadruplexes. Arrows at the bottom of average plots indicate approximate positions of histone genes. According to the online tool QGRS mapper (Quadruplex forming G-Rich Sequences), there are 165 potential G-quadruplex forming sequences across the entire *Drosophila melanogaster* histone repeat unit using the following parameters: QGRS Max Length: 30 | Min G-Group Size: 2 | Loop size: from 0 to 36 | overlaps included. (**C**) UCSC browser track snapshot of CUT&Tag data targeting RNA Pol II Ser5P at Dodeca-satellite repeats. (**D**) UCSC browser track snapshot of CUT&Tag data targeting RNA Pol II=Ser2P at Dodeca-satellite repeats. Black lines below the browser tracks indicate location of Dodeca-satellite repeats. Data for (A) and (C) is merged data of 5 biological replicates for control, actinomycin D- and aclarubicin-treated samples and 6 biological replicates for doxorubicin-treated samples. Data for (B) is merged data of 3 biological replicates for control, doxorubicin- and aclarubicin-treated samples and 2 biological replicates for actinomycin D-treated samples. Data for (D) is merged data of 6 biological replicates for doxorubicin, actinomycin D- and aclarubicin-treated samples and 5 biological replicates for control samples.

Cell #s	control 0hr	control 24hrs	doxorubicin 0hr	doxorubicin 24hrs	actinomycin D 0hr	actinomycin D 24hrs	aclarubicin 0hr	aclarubicin 24hrs
cell # *10^6	1	2.3	1	1.16	1	0.86	1	1.5
cell # *10^6	1	5.62	1	1.373333	1	1.4	1	1.04
cell # *10^6	1	2.9	1	1.25	1	1.2	1	1.09

Table. S1. Cell growth after 24 hours in each drug treatment One million cells seeded at timepoint 0 in each conditioned. Cell number measured after 24 hours.

24-hour cell size	Control	Doxorubicin	Actinomycin D	Aclarubicin
Cell size 1	9.75	9.8	8.75	8.59
Cell size 2	9.7	10.4	8.98	8.9
Cell size 3	9.69	11.3	8.99	8.84
Cell size 4	9.9	10	8.35	8.43
Cell size 5	9.75	10.1	8.07	8.31
Cell size 6	9.6	10.2	8.07	8.12
Cell size 7	9.9	10.3	8.07	8.1
Cell size 8	9.8	10.5	9.4	9.2
Cell size 9		10.2		
Cell size 10		10.5		
Cell size 11		10.8		

Table. S2. Average cell size after 24 hours drug treatment.

1-hour cell size	Control	Doxorubicin	Actinomycin D	Aclarubicin
Cell size 1	9.8	9.7	9.6	9.8
Cell size 2	9.81	10	9.8	9.73
Cell size 3	9.65	9.8	9.77	9.69

Table. S3. Average cell size after 1 hour drug treatment.

Supplementary excel file 1: Excel file containing all *Drosophila melanogaster* promoters sorted in descending order by distance to closest upstream neighboring promoter elements.

Supplementary excel file 2: Excel file containing all *Drosophila melanogaster* promoters cluster via k-means clustering (k = 3) on CUTAC data shown in Fig. 2.

Supplementary excel file 3: Excel file containing all *Drosophila melanogaster* divergent promoters sorted in descending order by distance to closest upstream neighboring promoter elements.

Supplementary excel file 4: Excel file containing all *Drosophila melanogaster* tandem promoters sorted in descending order by distance to closest upstream neighboring promoter elements.
